# Microglia cause HIV-induced transcriptional and metabolic changes in human neural organoids

**DOI:** 10.1038/s42003-026-09864-9

**Published:** 2026-03-19

**Authors:** Pamela E. Capendale, Leanne C. Helgers, Anoop T. Ambikan, Renata Vieira de Sá, Katja C. Wolthers, Teunis B. H. Geijtenbeek, Adithya Sridhar, Ujjwal Neogi, Dasja Pajkrt

**Affiliations:** 1 OrganoVIR Labs, Department of Pediatric Infectious Diseases, Emma Children’s Hospital, Amsterdam Institute for Reproduction and Development, Amsterdam Institute for Infection and Immunity, Amsterdam UMC, location Academic Medical Center, University of Amsterdam, Amsterdam, The Netherlands; 2 OrganoVIR Labs, Department of Medical Microbiology, Amsterdam Institute for Infection and Immunity, Amsterdam UMC, location Academic Medical Center, University of Amsterdam, Amsterdam, The Netherlands; 3 Department of Experimental Immunology, Amsterdam UMC Location University of Amsterdam, Amsterdam, The Netherlands; 4 Amsterdam institute for Infection and Immunity, Amsterdam, The Netherlands; 5 The Systems Virology Lab, Division of Clinical Microbiology, Department of Laboratory Medicine, ANA Futura, Karolinska Institutet, Stockholm, Sweden; 6 Department of Research & Development, UniQure Biopharma B.V., Amsterdam, The Netherlands; 7 Emma Center for Personalized Medicine, Amsterdam UMC, Amsterdam, The Netherlands

**Keywords:** Systems virology, Retrovirus

## Abstract

Human immunodeficiency virus (HIV) can invade the central nervous system during the initial stages of infection and contribute to HIV-associated neurocognitive disorder, affecting up to 50% of people living with HIV (PLWH). To investigate HIV-1–induced immunometabolic changes in the brain, we used a three-dimensional microglia-embedded human neural organoid model. Transcriptomic analysis and genome-scale metabolic modeling revealed that HIV-1 infection led to more pronounced transcriptional changes in the presence of microglia, including upregulation of pro-inflammatory pathways. We identified CCR6, important for HIV-1 permissiveness, to be significantly upregulated upon infection. Metabolic analysis showed increased expression in metabolite transport-related genes, including solute carrier (SLC) genes and altered amino acid metabolism, particularly involving arginine, proline, and tyrosine. These microglia-driven immunometabolic changes may contribute to neuronal dysregulation and, subsequently, neurological complications, which are often observed in PLWH. Early detection of these alterations could support timely therapeutic intervention to improve HIV-related neurologic insult.

## Abstract (web summary)

In neuronal organoids, HIV infection drives alternative metabolic pathways to meet increased energy demands, altering metabolite transport and amino acid metabolism while activating microglial glycolysis, inflammatory responses, and astrocyte activation.

## Introduction

Human immunodeficiency virus type 1 (HIV-1) infection remains a global health burden. In untreated individuals, infection may result in neurological complications collectively known as HIV-associated neurocognitive disorders (HAND). Moreover, these neurocognitive disorders can persist despite combination antiretroviral therapy (cART), and persistent impairment most often manifests as asymptomatic neurocognitive impairment (ANI) or mild neurocognitive disorder (MND), with HIV-associated dementia being rare. HAND affects the quality of life in nearly half of people living with HIV (PLWH)^1–4^. Microglia, the resident immune cells of the central nervous system (CNS), are susceptible to HIV infection and can function as a latent HIV reservoir^5,6^. Eradication of latent HIV-infected cells is a significant hurdle, as HIV integrates into the host genome and microglia have a long-lived nature with limited replenishment^7,8^.

A persistent HIV reservoir can result in the expression of HIV proteins, low levels of viremia, and immune activation, leading to a chronic inflammatory microenvironment in the brain^4^. This can cause a shift in metabolic interplay between cells, resulting in a lack of metabolic support to neurons and thus loss of neuronal function, slowly evolving into impaired cognitive function^9–11^. Previous studies have reported alterations of the cellular metabolism in PLWH compared to healthy controls despite effective long-term cART^12,13^. Indeed, results from metabolomics and proteomics analyses on the plasma of PLWH showed a link between metabolic abnormalities and alterations in markers of neurological diseases^4,14^. These findings indicate that CNS metabolism is linked to neuroinflammation that may lead to HIV-induced neurological injury.

To better understand how metabolic dysregulation contributes to neuroinflammation in the context of HIV, studying the initial stages of infection in the CNS is essential, yet challenging due to sampling the CNS. Neural organoids with integrated microglia provide an opportunity for studying HIV-induced alterations in neural tissue in the CNS. In this study, we established a co-culture model using human induced pluripotent stem cell (hiPSC) derived microglia and neural organoids from three distinct hiPSC lines. First, microglia were infected with HIV-1_JR-CSF_, a CCR5-tropic molecular clone from a primary isolate from the CSF of an HIV-infected patient with severe HAND^15–17^. Next, (infected) microglia were co-cultured with dorsal forebrain organoids, herein referred to as neural organoids, resulting in integration of microglia. By using transcriptomics and genome-scale metabolic modeling, we study transcriptomic changes of HIV-infected microglia embedded neural organoids (MNO) and their impact on the immunometabolic alterations in the human neural microenvironment.

## Results

### HIV-1_JR-CSF_ infected microglia integrate into neural organoids and transfer viral particles to astrocytes, not neurons

**Fig. 1:**
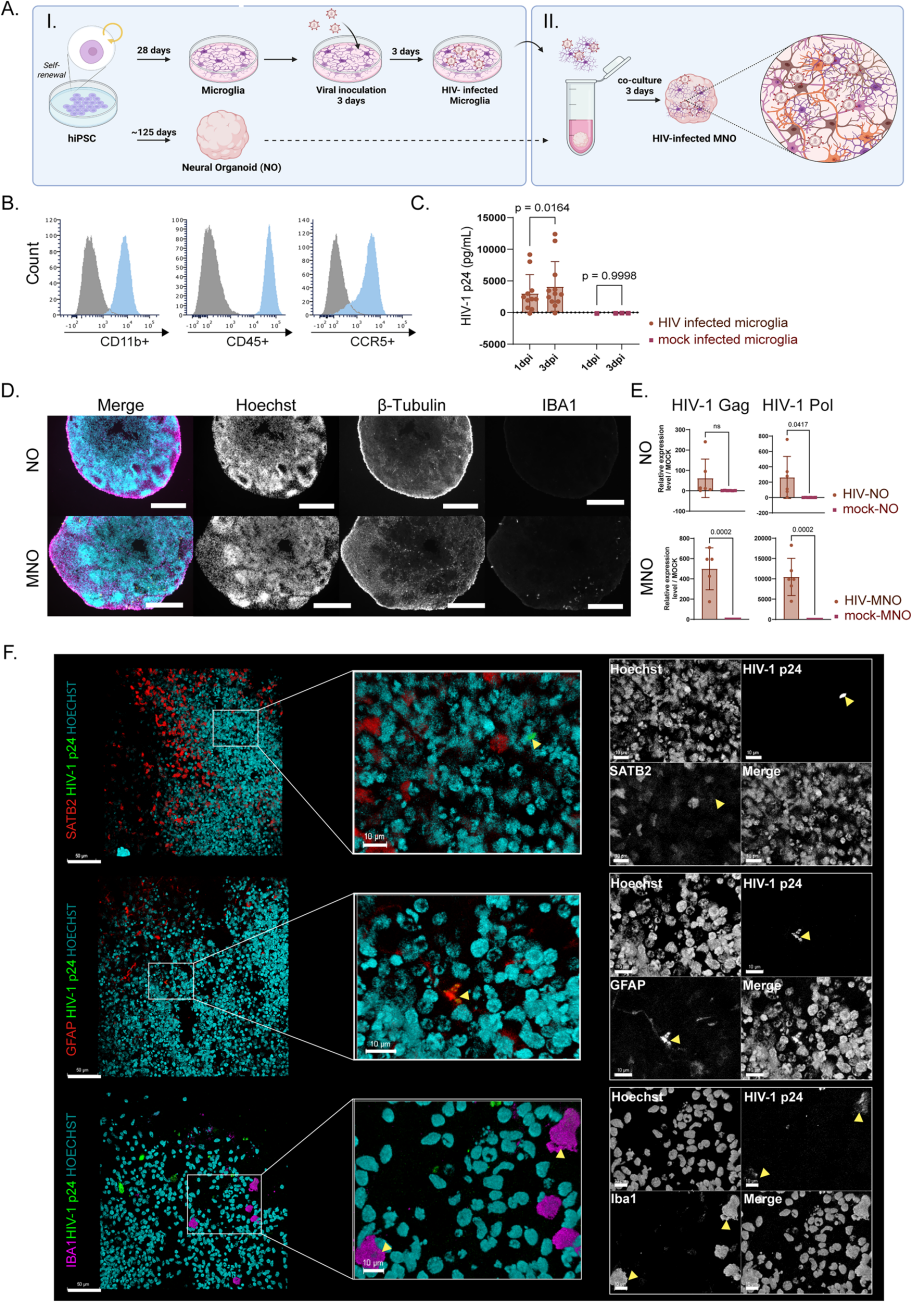
HIV-1_JR-CSF_ replicates in microglia and integrates into neural organoids. **A** Schematic overview of the generation of HIV-infected microglia and co-culturing of microglia and neural organoids to generate MNO. **B** Marker expression of microglia validated by flow cytometry for CD11b + , CD45+, and CCR5+ (blue) compared to unstained control (grey). **C** Quantification by ELISA of intracellular HIV p24 levels in microglia at one and three dpi (pg/mL). **D** Immunocytochemistry imaging confirmed the integration of microglia in neural organoids observed after 3 days of co-culturing. Scale bar = 300 µm. **E** HIV Gag and Pol transcript levels quantified by RT-qPCR in NO and MNO three days after co-culture with either HIV-infected or mock-infected microglia. Gene expression was normalized using reference genes and the 2^−ΔΔCT^ method, and data were visualized as a fold change to mock. **F** Immunofluorescence staining of neural organoids embedded with HIV infected microglia at 3 days post-co-culturing, showing the colocalization of HIV p24 in astrocytes (GFAP+) and microglia (IBA1+), but not neurons (SATB2+). Scale bar = 50 µm (left panels) and 10 µm (middle and right panels). In all cases, data correspond to the mean ± SD of two or more technical replicates (individual organoids) in three biological replicates (hiPSC line) in independent experiments. Statistical significance was determined using an unpaired two-tailed *t* test, *P  <  0.05. dpi days post-infection, HIV HIV-1, NO neural organoids, MNOs neural organoids embedded with microglia, mock- mock-infected, HIV- HIV infected, HIV HI- inoculated with heat-inactivated HIV. Created with BioRender.com.

As a model to study the impact of HIV on the CNS and the role of metabolic reprogramming due to infection, we generated regionalized neural organoids with a forebrain identity, hereafter called neural organoids (NO) and co-cultured with HIV-1_JR-CSF_ infected microglia (HIV-MNO) or mock infected microglia (mock-MNO) (Fig. 1A). Microglia embedded in neural organoids with a forebrain identity were chosen as a model as the HIV-1_JR-CSF_ strain is a viral isolate that preferentially infects microglia and perivascular macrophages in the CNS. The frontal cortex is a brain region that is rich in microglia, and also associated with HIV-associated neurocognitive disorders based upon human postmortem tissue and animal model studies^18,19^. First, microglia were generated from three distinct hiPSC lines. To validate microglial identity (CD11b+, CD45+), flow cytometry was performed. In addition, all three hiPSC lines showed expression of CCR5, the predominant microglial chemokine receptor essential for the susceptibility of hiPSC-derived microglia to HIV_JR-CSF_ (Fig. 1B and Supplementary Fig. 1)^20,21^. Next, microglia were inoculated with HIV-1_JR-CSF_ at 0.5MOI for three days. Successful infection of the microglia was confirmed as intracellular HIV-1 p24 levels were readily detected one day after inoculation and increased progressively three days post-inoculation, indicating HIV replication (Fig. 1C). These results were consistent results across donors as we observed on increase in p24 at 3 dpi (Supplementary Fig. 2). Subsequently, integration of (HIV infected) microglia in NO was accomplished by co-culturing for three days and affirmed through immunocytochemistry imaging and RT-qPCR of viral proteins (Fig. 1D, E). Neural organoids (without embedded microglia) were infected with HIV (HIV-NO) to distinguish the role of microglia during HIV infection in neural organoids. As expected, transcriptomic levels of HIV-1 proteins gag and pol were more abundant in HIV-MNO than HIV-NO, which underscores the pivotal contribution of microglia in replication and rapid establishment of the HIV reservoir in the CNS (Fig. 1E). Immunocytochemistry imaging showed a colocalization of HIV-1 p24 with microglia (IBA1+) and astrocytes (GFAP+), but not with neurons (SATB2+) (Fig. 1F). In both HIV-MNO and HIV-NO, HIV-1 protein trans-activator of transcription (tat) protein was shown outside of IBA1 colocalization in close proximity to neuronal markers (β-tubulin) which possibly indicates integration of Tat within the nucleus of neurons (Supplementary Fig. 3). This is as expected and in line with previous publications, as tat is a gene regulator which causes the protein to be taken up by cells and distribute more widely compared to p24, which is primarily found in cells during HIV-1 replication. Together, these results show that infectious HIV-1_JR-CSF_ infection was established in hiPSC-derived microglia, which were subsequently successfully integrated into neural organoids, and viral proteins were transferred into neighboring cells.

### Microglia are essential for HIV-induced transcriptional changes in neural organoids

**Fig. 2:**
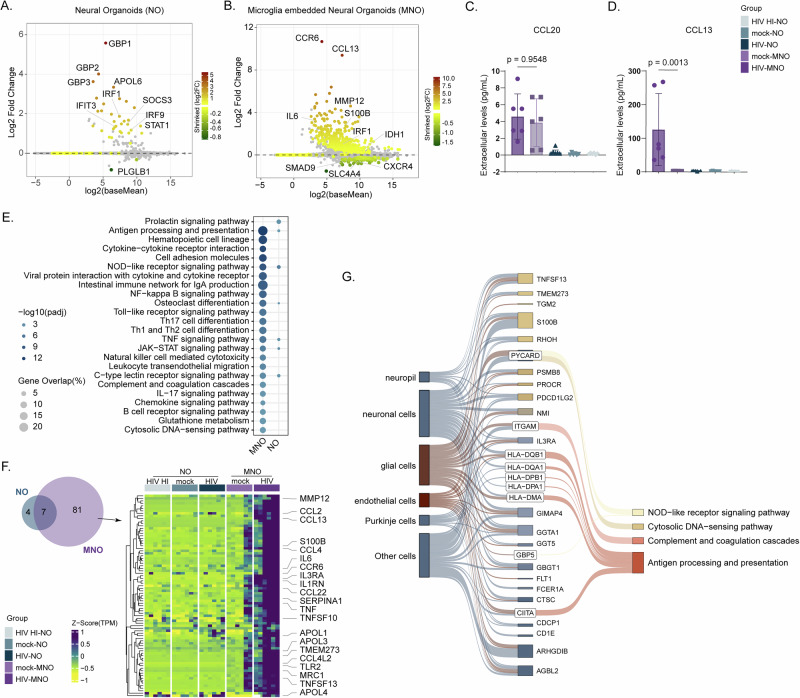
Microglia are essential for HIV-induced transcriptional changes in neural organoids. MA plot representing DEGs comparing HIV infection vs mock infection in **A** NOs and **B** MNOs (adjusted *p* < 0.05), bar graphs representing extracellular cytokine levels of **C** CCL20 and **D** CCL13 in supernatant of HIV-MNO, mock-MNO, HIV-NO, mock-NO and HIV HI-NO. Data were analyzed with a one-way ANOVA followed by Tukey’s post-hoc test (**E**) pathway analysis showing significantly altered pathways (adjusted *p* < 0.05) comparing HIV infection vs. mock infection in NOs and MNOs, **F** Heatmap representing significantly altered genes with high expression changes (adjusted *p* < 0.05 and L2FC > |2|) comparing HIV infection vs mock infection in NOs (*n* = 11) and MNOs (*n* = 88) representing two samples from three donors in the columns, **G** Sankey plot showing associations between significantly regulated genes with high expression changes (adjusted *p* < 0.05 and L2FC > 2) comparing HIV infection vs mock infection and their respective cell type and pathways. Data represent two technical replicates for three batches using three individual hiPSC donors. HIV HIV-1, NO neural organoids, MNOs neural organoids embedded with microglia, mock- mock infected, -HIV HIV infected, -HIV HI inoculated with heat-inactivated HIV.

To investigate the cellular and molecular mechanisms underlying HIV-induced neuroinflammation in the brain, we conducted RNA-seq to assess how HIV infection alters the transcriptome of neural organoids. RNA-seq analysis revealed significant transcriptional changes in both HIV-MNO and HIV-NO compared to uninfected controls, mock-MNO and mock-NO, respectively. Transcriptional changes were more abundant in the presence of microglia, where HIV infection in MNO caused 329 genes to be differentially expressed (DEGs) (adjusted *p* < 0.05 & log2fold change (LFC)2FC > 0.5), compared to 33 DEGs in NO (Fig. 2A, B). We identified the chemokine receptor CCR6 to be upregulated in MNO upon HIV infection. We measured extracellular levels of CCL20, the only known ligand of CCR6^22^, where no significant increase was observed in the supernatant between HIV-MNO and mock-MNO (Fig. 2C). Another gene that was upregulated was chemokine ligand CCL13, of which significantly increased extracellular levels were confirmed in HIV-MNO (Fig. 2D). Transcriptome levels of S100B and GFAP were significantly upregulated in HIV-MNO, a hallmark of astrocytosis^23^, as compared to no significant difference in HIV-NO (S100B adjusted *p* = 5.06E-07, GFAP adjusted *p* = 0.0109611844). Pathway enrichment analysis of the significantly regulated genes showed that a plethora of pathways were elevated in HIV-MNO (23 pathways, adjusted *p* < 0.05), whereas only seven pathways were altered in HIV-NO (Fig. 2E). The most significantly altered pathways in HIV-MNO were related to inflammation (e.g., cytokine–cytokine receptor interaction, antigen processing and presentation, viral protein interaction with cytokine and cytokine receptors, and JAK-STAT and TNF signaling pathways). Furthermore, genes uniquely regulated in HIV-MNO with high expression changes (adjusted *p* < 0.05 & L2FC > 2) were analyzed for their expression profiles and cellular locations in the brain based on evidence from the human protein atlas^24^. The trend of this response was consistent for MNOs from all three donors, which can be observed in the heatmap (Fig. 2F). Upregulation of immune-related pathways was also observed, although to a lower extent, in the absence of microglia. To address the potential effects of HIV independent of viral replication, a heat-inactivated HIV control (HIV HI-NO) was included, which showed similar transcriptional changes to mock-NO (Fig. 2F).

The cell types involved are displayed via a Sankey plot, showing associations between DEGs in HIV-MNO and their respective cell types and pathways (Fig. 2G). As expected, many DEGs are associated with glial cells, including microglia, astrocytes, and neuronal cells. Taken together, these results highlight the importance of the inclusion of microglia in recapitulating HIV-induced neuroinflammation.

### Metabolic modeling shows changes in metabolic flux induced by HIV-infected microglia

**Fig. 3:**
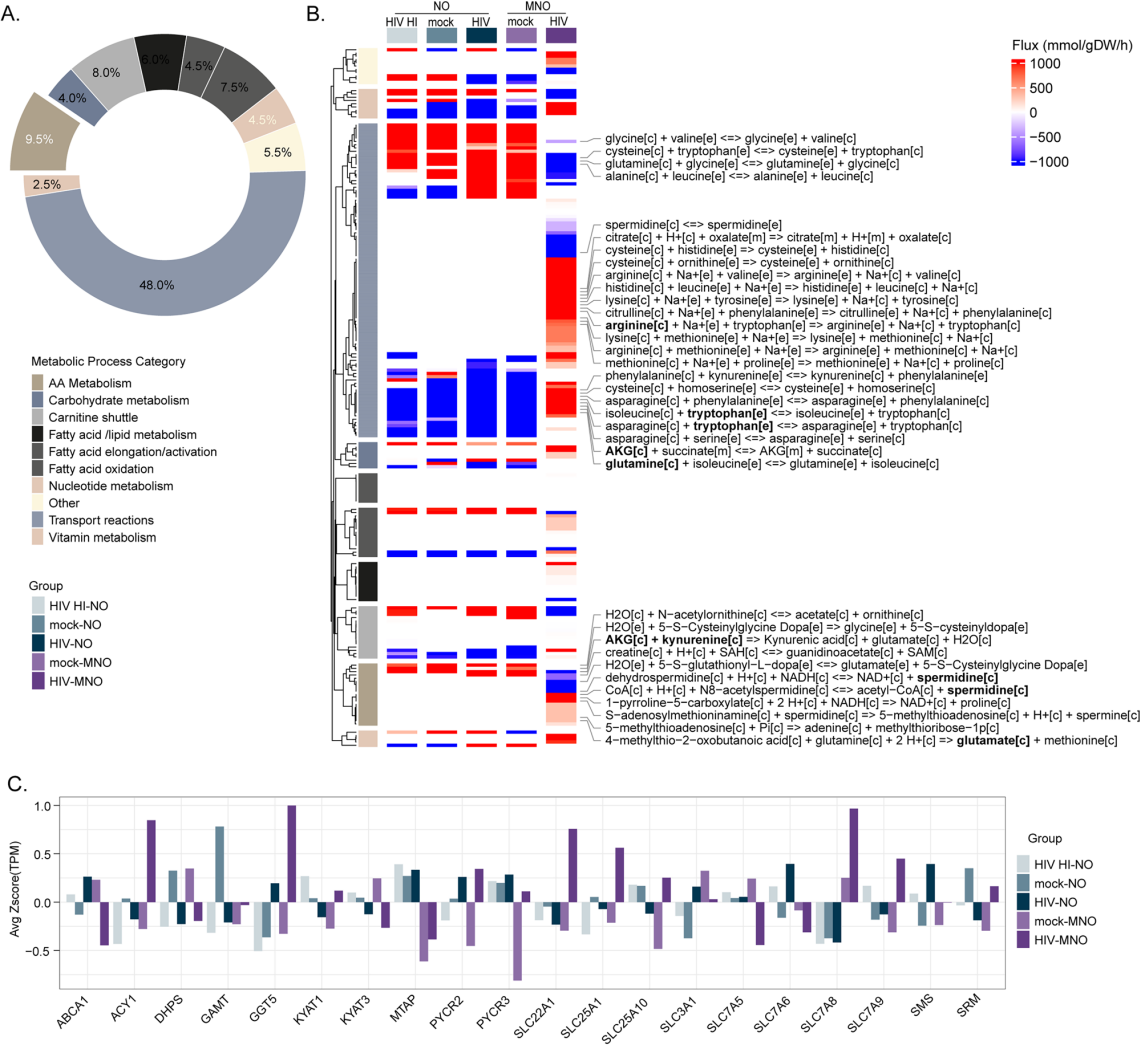
Changes in metabolic flux induced by HIV-infected microglia. **A** Percentage of changes observed in various metabolic process categories based on the number of metabolic reactions with altered flux in HIV-MNO compared to other groups. **B** Heatmap displaying predicted flux values of metabolic reactions uniquely altered in HIV-MNO compared to other groups. **C** Average expression level of metabolic genes based on the RNA-seq data among different sample groups. Data represent two technical replicates for three batches using three individual hiPSC donors. [c] refer to cellular, [e] refer to extracellular, [m] refer to mitochondria. HIV HIV-1, NO neural organoids, MNOs neural organoids embedded with microglia, mock- mock infected, HIV- HIV infected, HIV HI- inoculated with heat-inactivated HIV.

Context-specific metabolic network modeling and flux balance analysis (FBA) were performed to identify metabolic reactions altered in response to HIV-1 infection in human neural organoid models. Gene expression data were used to contextualize the human generic metabolic network model to represent each of the organoid models used in the study. GEM contextualization resulted in models with a number of reactions 8959, 8916, 8757, 8763, and 8761, respectively, for HIV-MNO, mock-MNO, HIV-NO, mock-NO, and HIV HI-NO. FBA was conducted to predict the metabolic turnover rate of any reaction in the model, identifying HIV-induced alterations in MNO. The analysis identified 200 specific reactions in HIV-MNO, primarily belonging to the categories of metabolite transport reactions and amino acid metabolism (Fig. 3A, B). Reactions related to metabolite transport and amino acid metabolism were further analyzed for their activity. The results revealed the export of arginine from the cytoplasm and accumulation of tryptophan within the cytoplasm. Additionally, α-ketoglutarate (AKG) was transported from the cytoplasm to mitochondria, along with increased glutamate production in the cytoplasm. Furthermore, the reaction producing AKG and kynurenine from glutamate and kynurenic acid was found to be active in HIV-MNO. Overall, a higher production of AKG in the cytoplasm and its subsequent transport to the mitochondria was specifically observed in HIV-MNO as compared to other groups (Fig. 3B). Additionally, the expression of catalytic genes corresponding to the reactions labeled in Fig. 3B was analyzed for their uniqueness in HIV-MNO. These catalytic genes were identified based on the gene-protein reaction (GPR) rules from the generic model of the human metabolic network. The analysis revealed that metabolic genes, particularly members of the solute carrier (SLC) family such as SLC25A10, SLC25A1, SLC22A1, SLC7A8, and SLC7A9 exhibited comparatively higher expression in HIV-MNO as compared to the other groups (Fig. 3C). These genes play a crucial role in transporting key metabolites such as α-ketoglutarate (AKG), succinate, citrate, oxalate, cysteine, ornithine, histidine, phenylalanine, kynurenine, and spermidine between cellular compartments. Furthermore, the catalytic genes PYCR2, ACY1, and GGT5 also showed increased expression in HIV-MNO. These genes are involved in catalytic functions within the arginine, proline, and tyrosine metabolism pathways, highlighting potential metabolic alterations associated with HIV-MNO. Taken together, context-specific metabolic modeling and FBA revealed distinct HIV-induced metabolic rewiring in MNOs, characterized by enhanced amino acid metabolism and mitochondrial metabolite transport. These findings highlight the importance of microglia in shaping the metabolic landscape of the HIV-infected brain microenvironment.

## Discussion

Despite cART treatment, neurological complications are still a burden for many PLWH. In this study, we expanded current knowledge on HIV-induced immunometabolic dysregulation by employing a fully hiPSC-derived microglia–neural organoid model. Our findings reveal that HIV-infected microglia play a central role in driving neuroinflammation and metabolic reprogramming. Specifically, we observed upregulation of CCR6 signaling, astrocyte activation, and disrupted tryptophan and polyamine metabolism, all of which mirror features of HIV-associated neuropathology. Identifying such early metabolic alterations may ultimately guide the development of biomarkers for HAND risk stratification and enable timely intervention.

Recent studies comparing microglia models for understanding the pathobiology of HIV-1-infected microglia confirm that hiPSC-derived microglia and primary microglia provide a more representative microglial culture model for HIV research compared to macrophages or microglial cell lines^25,26^. Several different microglia-containing neural organoids have been used previously by multiple groups and have been shown to support productive HIV infection and HIV-induced inflammatory responses^26–30^. In line with our results, these studies underscore the importance of the inclusion of microglia in recapitulating HIV-induced neuroinflammation, although the data linking to immunometabolic dysregulation is limited. In a related study, Dos Reis et al. demonstrated that both immortalized and primary microglia can be successfully infected and integrated into NPC-derived brain organoids, supporting viral replication and altering cytokine expression profiles^27^. However, in our study, we employed a fully hiPSC-derived model in which both microglia and neural organoids originate from the same donor, which provides a unique platform for investigating patient-specific aspects of HIV-1 neuropathogenesis and microglial responses.

Reviews on HIV-1 tropism in the CNS have consolidated evidence identifying microglia and perivascular macrophages as the primary target cells^5,6^. In line with our observations, a study using neural organoids showed colocalization of HIV markers with microglia, and (limited) colocalization with astrocytes^29^. On the contrary, our observation of late-stage viral proteins (p24) in astrocytes contrasts with a previous study using neural organoids, which observed an absence of co-immunostaining of HIV gag and astrocyte markers^27^. Permissiveness of astrocytes is still a topic under debate, as HIV nucleic acid and proteins can be found in astrocytes, potentially due to engulfment or expression of early viral proteins^31^. However, productive astrocyte HIV infection is thought to be restricted due to the absence of late viral proteins in astrocytes of post-mortem tissue of PLWH with HAND^23^.

Our data showed that microglia are essential for inducing HIV-related transcriptional differences in neural organoids, as we identified several genes (amongst others, CCR6) to be importantly upregulated. CCR6 is a (weak) co-receptor for HIV entry and is of significance in the context of HIV permissiveness^22^. Some untreated PLWH naturally control HIV infection, resulting in a limited HIV reservoir, even compared to PLWH on cART. Recently, it was found that this population with natural immunity shows reduced CCR6 expression^32^. Previous organoid studies using neural organoids identified increased expression of CXCR4 and CCR5 upon HIV infection, but to our knowledge, CCR6 upregulation has not been reported before^29^. Furthermore, CCR6, together with its only ligand CCL20, influences HIV entry. Interestingly, although plasma levels of the CCR6 ligand CCL20 are elevated in PLWH, we did not observe extracellular CCL20 induction in HIV-MNO^33,34^. CCR6 upregulation in the absence of CCL20 elevation may indicate ligand-independent signaling by activating the receptor through interactions with other ligands like human beta-defensins^35^. This alternative route can lead to enhanced cell migration and activation, which has been reported to modulate inflammatory cascades^36,37^. Given CCR6’s dual role in viral entry and immune regulation, the underlying mechanisms of CCR6 upregulation in HIV infection would be interesting to study in more detail to better understand its potential role in modulating HIV progression and inflammatory immune responses. In addition, further studies need to elucidate the usage of CCR6 as a co-receptor of HIV in the CNS, which will help to define its role in the establishment of an HIV reservoir and HAND.

Our results show that HIV infection induces interferon (IFN)-associated pathways specifically in the presence of microglia. This aligns with observations from post-mortem HIV-infected brain tissue^38^ and plasma samples^39^ and previous organoid studies^28,38^, which consistently report heightened IFN responses during HIV neuropathogenesis. Notably, microglia-embedded organoid models using single-cell RNA-seq have shown that HIV-infected microglia drive IFN signaling predominantly in astrocytes as a bystander effect^38^. Moreover, an IFN-responsive microglial subtype has been linked to neurotoxicity through aberrant synaptic pruning, excessive cytokine production, and dysregulated phagocytosis^40^. Therefore, our findings are in line with these established mechanisms and pathological features of HAND, and previous findings based on organoid work.

The observed upregulation of cytokines and chemokines, including CCL13, TNF-α, and IL-1β and markers associated with astrocyte activation and neuroinflammation, including GFAP and S100B, is in line with previous neural organoid studies^27,28,30,41^ and previously suggested to be associated with HAND pathology^28,42,43^. Pathway enrichment analysis showed that HIV infection in MNO results in a plethora of altered pathways, of which many are related to (virus-induced) inflammation. The trend of this response was consistent for MNOs from all three hiPSC donors, pointing towards a conserved mechanism behind the HIV-induced transcriptomic changes in immune pathways. This is not surprising, as neuroinflammation is a key feature associated with HIV neuropathology^44^. The upregulation of immune-related pathways was observed to a lower extent in the absence of microglia. The transcriptomic changes can thus be attributed to the presence of microglia, which not only serve as a susceptible target to allow for an increase in viral particles over time but also for an enhancement of the observed immune response. As expected, cell type association analysis showed many DEGs upon HIV infection in MNO to be associated with both glial, including microglia and astrocytes, and neuronal cells.

Analyzing metabolic fluxes by FBA on a genome-wide scale is a novel and effective tool in the development of GEM, which has previously been used to predict anti-viral targets, including for HIV^45^. We predicted glutamine uptake and conversion to glutamate, along with an increased cytoplasmic production of AKG and its transport to mitochondria in HIV-MNO. This profile fits the metabolic profile of cells that are looking for alternative energy sources, other than glucose, upon metabolic stress. In addition, the altered arginine and tryptophan metabolism in HIV-MNO suggests significant metabolic reprogramming associated with HIV infection. Consistent with this, the upregulation of SLC7A8 and SLC7A9 (transporters of neutral amino acids including cysteine, tryptophan, leucine, and phenylalanine) supports increased substrate availability for glutamate synthesis, glutathione production, and kynurenine pathway activity. These changes may contribute to neuroinflammation by promoting oxidative stress and immune activation, potentially exacerbating HIV-associated neuropathology^46^. Additionally, disruptions in energy metabolism could impair cellular homeostasis^46^, a process further supported by increased expression of mitochondrial carriers SLC25A1 and SLC25A10, which mediate citrate and AKG exchange and therefore influence TCA cycle activity and redox balance. While excess glutamate production increases the risk of excitotoxicity, a key factor in neurodegeneration^47^ the observed SLC expression pattern aligns with enhanced glutamine–glutamate cycling and mitochondrial metabolite transport that could exacerbate this vulnerability. Altered tryptophan metabolism may further impact neurotransmitter balance, affecting serotonin and kynurenine pathway metabolites, which are linked to cognitive dysfunction^48^, and the upregulation of SLCA8 and SLC7A9 involved in tryptophan handling further supports this metabolic shift. Therefore, our data predicted that HIV infection impacts metabolic reactions in MNO, particularly in metabolite transport and amino acid metabolism, highlighting a metabolic rewiring upon HIV infection and potentially contributing to neuroinflammatory and neuropathogenic mechanisms.

One link between inflammation and altered metabolic flux is the need to increase cellular metabolic activity in response to viral infection. Many HIV-associated immune stimuli drive immunometabolic dysfunction, e.g., HIV-induced inflammation leading to cellular alterations in metabolic behavior. The metabolites that have previously been associated with immune activation and disease progression in PLWH are metabolites belonging to carbohydrate and amino acid metabolism^49,50^. Our results are aligned with these reports as we predicted the export of arginine from the cytoplasm, accumulation of tryptophan in the cytoplasm, and transportation of AKG from the cytoplasm to mitochondria, correlating with increased glutamate production. Notably, the presence of an active reaction converting glutamate and kynurenic acid into AKG and kynurenine within HIV-MNO underscores how amino acid metabolism can importantly shift in response to HIV-induced inflammation. Tryptophan serves as a critical amino acid in this context, being primarily metabolized through the kynurenine pathway during inflammatory responses due to the activity of indoleamine 2,3-dioxygenase (IDO) and tryptophan 2,3-dioxygenase (TDO)^51,52^. This metabolic pathway accounts for approximately 90–95% of tryptophan degradation, thus reducing its availability for serotonin production, which is essential for various physiological functions, including mood regulation and immune responses^53^. Recently, a multi-omics study reported the profound impact of dysregulated tryptophan metabolism via the kynurenine pathway in immunometabolically complicated HIV-infected patients on long-term cART^4^. This dysregulation can disrupt amino acid balance and energy metabolism, leading to elevated levels of serotonin, kynurenate, and quinolinate in response to an inflammation driven by tryptophan breakdown. Moreover, this chronic inflammatory microenvironment may contribute to synaptic dysregulation ex vivo, as reported recently, heightening the risk of neurological and psychiatric symptoms by perturbing neuroimmunometabolic processes. The interplay between tryptophan, kynurenine, glutamate, and AKG indicates that metabolic pathways are indeed dynamically regulated in response to inflammatory stimuli, potentially driving alterations in systemic homeostasis that have broad implications for the understanding of inflammatory diseases^51,54^.

**Fig. 4:**
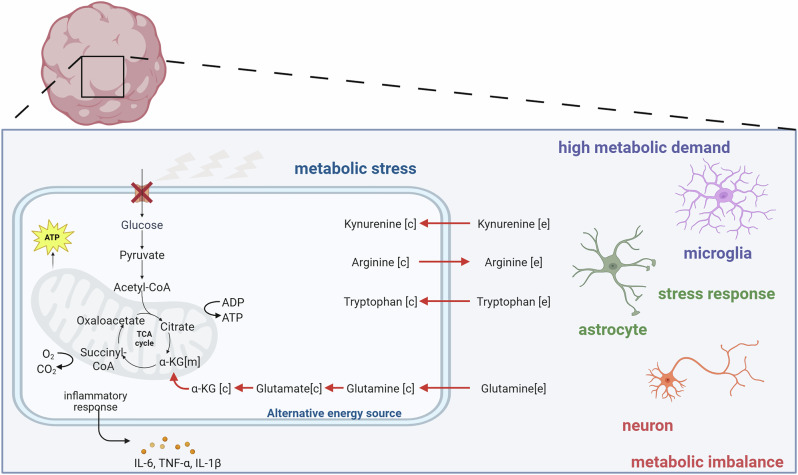
Immunometabolic imbalance caused by HIV infection in the brain microenvironment. Our data shows changes in metabolite transport and amino acid metabolism upon HIV-1 infection of microglia-embedded neural organoids. These observed changes suggest that HIV-1 infection causes cells to use alternative energy routes to account for the high metabolic demand, which can contribute to a loss of metabolic support to neurons, and an inflammatory microenvironment.

How microglia-associated metabolic shifts contribute to HAND, however, remains enigmatic. Our results suggest that, besides inflammatory responses, HIV infection causes cells to use alternative routes to account for the high metabolic demand—evidenced by the altered metabolite transport and amino acid metabolism (Fig. 4). The persistence of HAND despite cART highlights the urgency of identifying the factors underlying pathogenesis caused by these HIV reservoirs^55^. Importantly, metabolic health should be considered alongside viral suppression when aiming to improve long-term health outcomes for PLWH. As we focus on the early stage of HIV-1 infection in the CNS in this work, it would be of interest to look into chronic effects using longer-term studies in future work to elucidate how the metabolic shifts identified in our work contribute to HAND over time.

### Limitations

Although MNO has provided a relevant way to study HIV neuropathology, some limitations are still present. First, the CNS cellular complexity and cellular composition differ per cellular compartment and even brain region. In our model, the dorsal forebrain organoids do not represent the full complexity and physiological consequences of a real-life-sized human brain.

HIV infection does not result in a uniform immunologic and metabolic response of different cells within the brain. Changes in cell-specific and often opposing metabolic profiles during HIV infection are therefore hard to distinguish. This complicates studying metabolic changes in the CNS. In this study, RNA-seq was performed in bulk; however, to get more specific insights into the contributions of each cell in this complex metabolic network, single-cell sequencing is needed.

There are no oligodendrocytes present in our microglia-embedded NO model to promote the protection of neural axons by myelination and limit exposure to extracellular metabolites. Similar to astrocytes, oligodendrocytes are glycolytic and able to support neuronal metabolic demands by shuttling lactate and pyruvate to the neurons. Metabolic stress induces a prioritization of metabolites to cell survival rather than myelin maintenance, resulting in myelin loss and white matter damage, which can be observed in PLWH. To date, there is no evidence of HIV infection of oligodendrocytes in vivo, and the consequences of HIV infection on oligodendrocytes are likely to be indirect. However, given the above-mentioned facts, they do have their own role in the metabolic collaborative network to sustain homeostasis and thus play a role in HIV-induced neuropathology.

While our study focuses on transcriptional and metabolic changes, functional readouts such as calcium imaging or multielectrode array recordings would provide direct evidence of neuronal impairment. In addition, to strengthen the mechanistic insights derived from GEM and FBA, validation using metabolic flux assays (e.g., Seahorse XF analysis and LC-MS/MS metabolomics) would be beneficial. As our results are hypothesis-generating, we propose the inclusion of such functional validation of neuronal activity in follow-up studies to provide critical evidence of the influence of our observed inflammatory and metabolic changes from HIV infection and microglial activation on neuronal health. Additionally, for follow-up studies, it would be interesting to include a control with heat-inactivated HIV in the MNO condition, to be able to better differentiate between replication-dependent and independent effects of the virus in the presence of microglia. Other valuable directions for future research would be investigating inflammasome-associated activation driving my HIV by microglia specifically^44^, or the impact of cART on the metabolic pathways highlighted in this paper. Future possibilities include vascularization of neural organoids, which will allow for studying neuroinvasion. The permeability of the blood-brain-barrier (BBB) and behavior of BBB cell types are disrupted upon HIV infection, thereby contributing to the development of HIV neuropathogenesis.

## Methods

### Cell lines and viruses

PHA-stimulated human Peripheral Blood Mononuclear Cells (PBMCs) were isolated from buffy coats of healthy donors (Sanquin) by Lymphoprep (Axis-Shield) gradient and used to propagate HIV-1_JR-CSF_ stocks. HIV-1_JR-CSF_, a well-characterized molecularly cloned strain isolated from the CSF of an AIDS patient, was obtained from Dr. Irvin Chen through the NIH AIDS Reagent Program, Division of AIDS, NIAID^15,17^. Viral stock titration was performed using p24 ELISA (Perkin Elmer Life Sciences) and TZM-bl cells, which were obtained from John C. Kappes, Xiaoyun Wu, Birmingham, Alabama, USA and TranzymeInc., the NIH AIDS Reagent Program, division of AIDS, NIAID^56^. HIV-1_JR-CSF_ determined titer: 5,6 or 5,8 TCID50.

### Human induced pluripotent stem cell culture

Human induced pluripotent stem cells (hiPSCs) lines, IMR90 (IMR90-4/WISCi004-B, RRID:CVCL_C437, WiCell), M001 (SCTi002-A, RRID:CVCL_9S50, STEMCELL Technologies Inc.), and Gibco (TMOi001-A, RRID:CVCL_RM92, Gibco® Episomal hiPSC Line, Gibco®) were routinely cultured on 5 μg/mL human laminin 521 (Biolamina)-coated culture-treated six-well plates (CoStar®), maintained in mTeSR™ Plus (STEMCELL Technologies™) supplemented with 1% (v/v) Pen-Strep. Medium was refreshed daily, and stem cell lines were passaged weekly. Subculturing was performed weekly using ReLeSR™ (STEMCELL Technologies™) after removal of differentiated patches when necessary. The maintenance of the hiPSC lines was performed according to relevant guidelines and regulations.

### Generation of microglia

Using the STEMdiff Hematopoietic kit, hiPSCs were differentiated into hematopoietic progenitor cells (HPCs) in twelve days according to the manufacturer’s instructions. In short, hiPSCs are passaged as 100–200 μm aggregates into a Matrigel® (Corning) coated 12-well plate with aiming for 16–40 aggregates to be attached on day 1. From day three, mesodermal cells migrate outward from the colonies and on day 7, HPC emerge in suspension from the adhered colonies. HPCs were further differentiated into mature microglia using STEMdiff™ Microglia Differentiation Kit by adding half-medium volume of STEMdiff™ Microglia Differentiation medium every other day for 24 days. Quality control was performed by flow cytometry as recommended by the manufacturer, using CD45 PerCP Cy5.5 (#60018PS.1, STEMCELL Technologies™), Cd11b AF488 (#60040AD.1 STEMCELL Technologies™; Supplementary Fig. 1). CCR5 marker expression was confirmed using primary antibody CCR5 PE Clone 2D7 (#555993, BD Pharmingen).

### Generation of neural organoids (NO)

Neural organoids were generated using the STEMdiff™ Dorsal Foreneural Organoid Differentiation Kit (STEMCELL Technologies™). This protocol was chosen for its reported consistency in size and batch-to-batch variation^57^ and every batch of brain-region-specific organoids was routinely checked for the structural organization and cellular composition representative of the human forebrain by using immunocytochemistry and markers for expected cell types. The protocol was followed according to the manufacturer’s instructions with a modification, where, from day 26 on, neural organoids were placed on an orbital shake shaker (66 rpm) in a 37 °C incubator during culturing. After 43 days in culture, the neural organoids were maintained using STEMdiff™ Neural Organoid Maintenance Kit (STEMCELL Technologies™) with medium changes every two to three days until the organoids were used for further experiments at an age of ~125 days.

### Establishing a co-culture of HIV-1-infected microglia with NO (HIV-MNO)

Microglia were generated separately to allow combining them in controlled amounts at the start of the experiment. Microglia were counted to obtain 200.000 microglia per organoid and added into a Matrigel® (Corning) coated T75 culture flask containing STEMdiff™ Microglia Differentiation medium. HIV-1_JR-CSF_ stock at a titer of 0.5 TCID50 or mock inoculate was added to the flask and incubated for 72 h at 37 °C at 5% CO_2_. The mock control consisted of virus-free culture supernatant derived from the same preparation as HIV-inoculated samples, thereby controlling for background effects of the inoculation medium. Two days after infection of microglia, freshly prepared STEMdiff™ Microglia Differentiation medium was added to the flasks for cell maintenance. After 3 days of infection, the total volume of microglia suspension with HIV-1_JR-CSF_ or mock inoculate was collected into a 15 mL tube, and 1 mL containing 200.000 microglia was added per 15 mL tube. Each 15 mL tube was previously rinsed with Anti-Adherence Rinsing Solution (STEMCELL Technologies™) and contained one neural organoid in 3 mL Forebrain Organoid Maintenance (STEMCELL Technologies™) prior to microglia addition. The co-cultures were maintained for three days at 37 °C 5% CO_2_ incubator to ensure robust microglial integration and detection of early transcriptional and metabolic changes while avoiding loss of organoid viability. Co-cultures were washed upon collection with phosphate buffer saline (PBS, Lonza), centrifuged at 400 × *g* for 5 min, resuspended in 1 mL TRIzol™ Reagent and stored at −80 °C until further processing. For controls, the same procedure was performed without microglia, adding STEMdiff™ Microglia Differentiation medium cultured with HIV-1_JR-CSF_ viral inoculate (HIV-NO), heat-inactivated viral inoculate (HIV HI-NO) or mock inoculate (mock-NO). The heat inactivation of HIV inoculate was performed using a heat bath at 95 °C for 10 min.

### p24 antigen ELISA

To demonstrate replication of HIV-1_JR-CSF_ in microglia, intracellular p24 levels were measured in microglia at 1 day and 3 days post-infection. Cells were washed upon collection with PBS (Lonza) and spun down at 400 × *g* for 5 min. Supernatant was removed and resuspended in 80 µL PBS. Additionally, 10 μL Triton™ X-100 1% (v/v) (X100-5ML, Sigma-Aldrich) and 10 μL lysis buffer from the p24 ELISA kit (HIV Type 1 p24 Antigen ELISA, Zeptomatrix) to make a total of 100 μL sample and store at −20 °C O/N until further processing. Cell lysates were analyzed using the p24 ELISA kit (HIV Type 1 p24 Antigen ELISA, Zeptomatrix) according to the manufacturer’s protocol and a 1:10 dilution in Assay Buffer. Following the final incubation and addition of stop solution, the optical density of each well was read at 450 nm using an H1 Synergy plate reader (BioTek). The levels of p24 in the samples were quantified using a standard curve. A positive control and a negative control were taken along.

### RNA isolation

RNA isolation was performed by phase separation using TRIzol™ Reagent and PureLink™ RNA Mini Kit (Thermo Fisher Scientific), according to the manufacturer’s recommendations. In short, lysate with TRIzol™ Reagent was incubated at RT for 5 min to allow complete dissociation of nucleoprotein complexes. Per 1 mL TRIzol™ Reagent, 0.2 mL chloroform was added and shaken vigorously by hand for 15 s. The sample was centrifuged at 12,000 × g for 15 min at 4 °C. The upper phase containing RNA was transferred to a new tube, and an equal volume of 70% ethanol was added. The samples were vortexed and transferred to the Spin Cartridge to further proceed with the PureLink™ RNA Mini Kit (Thermo Fisher Scientific) according to instructions. Samples were eluted in 60 µl RNAse-Free Water and stored at −80 °C until used for RNA sequencing or RT-qPCR analysis.

### RT-qPCR

Equal volumes of eluted RNA were used for reverse-transcription using the SuperScript™ II Reverse Transcriptase synthesis kit (Thermo Fisher Scientific), and 5 μL of the cDNA was used for reverse-transcription quantitative PCR (RT-qPCR). RT-qPCR was performed on the CFX Connect Real-Time PCR Detection System (Bio-Rad) using software CFX Maestro 1.1. Transcription levels of target genes were quantified by the 2^−ΔΔCT^ method, normalization to the geometric mean of RPLP0 and RPLP2 as reference genes, as described previously^58^. Details on the used primers can be found in Supplementary Table 2.

### RNA sequencing (RNA-seq)

Human strand-specific mRNA sequencing was performed by Novogene, United Kingdom. In short, RNA isolated from organoids was used for RNA quality control, directional mRNA library preparation (poly A enrichment). Sequencing was performed using the Illumina Sequencing PE150.

### Flow cytometry analysis

Microglia cells were harvested and washed with PBS, followed by centrifugation at 300 × *g* for 5 min, and fixed and permeabilized using 250 µL of Cytofix/Cytoperm solution (BD, 554714) for 20 min at 2–8 °C. Cells were washed twice with 1 mL BSA Stain Buffer (BD, 554657), centrifuged at 300 × *g* for 5 min, and blocked using 2 µl FC-block (BD, 564220) per sample for 5 min at 2–8 °C. Cells were stained with a conjugated primary antibody CD11b-488 (60040AD.1, STEMCELL Technologies™), CD45 (60018PS.1, STEMCELL Technologies™) and CCR5 (CCR5 PE Clone 2D7, BD Pharmingen, 555993) at a dilution of 1:25 in a total volume of 25 µl and incubated for 30 min at 2–8 °C, followed by two washes with 1 mL BSA Stain Buffer (BD, 554657). Samples were resuspended in 100 µl BSA Stain Buffer and kept in the dark at 2–8 °C until analysis on the flow cytometer (BD FacsCanto ll). Background signal was determined using unstained controls. The gating strategy microglia flow cytometry analysis was as follows: debris in the sample was excluded by gating in the FSC-A SSC-A plot. Then, the single-cell population was determined using gating in the SSC-W and FSC-H plot. From here, positive populations were determined using a mock channel and the respective fluorescent channel of interest. Background signal was determined using unstained controls (Supplementary Fig. 4).

### Immunofluorescence staining

At the moment of harvesting, organoids were washed twice with 5 mL PBS and fixed in 4% (v/v) formaldehyde (Sigma-Aldrich) for 30 min at RT. The formaldehyde was removed, followed by two washes with 5 mL PBS. Dehydration of organoids was obtained by submerging them in 30% (w/v) sucrose (Merck) O/N 4 °C, and transferred to a Tissue-Tek® Cryomold®. Excess sucrose was removed as much as possible, and organoids were embedded in optimal cutting temperature compound (OCT, Tissue Tek) before being snap frozen using dry ice. Samples were stored at −80 °C until sectioning. Sections (20 µm) were made using a cryostat (NX71, Thermo Fisher Scientific) and collected on SuperFrost Plus slides (Thermo Scientific).

For immunostaining, sections were blocked for 1 h at room temperature (RT) in a blocking solution consisting of 10% (v/v) SeaBlock Blocking Buffer (Thermo Fisher Scientific) supplemented with 0.6% (v/v) Triton X-100 (Sigma) in PBS. Primary antibodies (Supplementary Table 1) were added in a 1:1 blocking solution:PBS and incubated O/N at 4 °C. Sections were washed three times with PBS for 5 min, and incubated with secondary antibody (Supplementary Table 1) solution and Hoechst (1:1000 in PBS, Thermo Fisher Scientific) at RT for 1 h. Samples were quenched using ReadyProbes Tissue Autofluorescence Quenching kit (Invitrogen, kit) and incubated for 5 min, followed by three PBS washes. Finally, slides were mounted with glass coverslips using ProLong Gold Antifade Mounting Medium (Invitrogen). NOs were imaged using a Leica TCS SP8-X microscope and Leica LAS AF Software (Leica Microsystems) or an EVOS M5000 microscope (Thermo Fisher Scientific). Z-stacks were also taken, and 3D reconstructions were made using the LAS-X 3D software (Leica Microsystems) and ImageJ 1.50I. Colocalization of different markers (supplementary Table 1) was assessed within the same biological replicates.

### Bioinformatics analysis

Raw transcriptomic sequencing data were processed using the nf-core RNA-seq v3.12.0 pipeline from the nf-core collection of community-curated, reproducible workflows^59^. Adapter trimming and quality filtering were performed using Trim Galore (integrated within nf-core RNA-seq), configured to remove standard Illumina adapter sequences. Reads were trimmed from the 3′ end where the Phred quality score fell below 20, and sequences shorter than 20 nucleotides after trimming were discarded. Paired-end read processing was applied where applicable, with both mates retained only if passing quality thresholds. High-quality reads were aligned to the human reference genome (GRCh38), obtained from the Ensembl genome database, using STAR v2.7.9a^60^. STAR was executed in two-pass mode with the following parameters: maximum intron length = 1,000,000 bp, minimum intron length = 20 bp, mismatch allowance = up to 10 mismatches per read, and outFilterMultimapNmax = 20 (reads mapping to more than 20 loci were discarded). Alignments were output in coordinate-sorted BAM format. Gene-level expression quantification was performed using Salmon v1.10.1 in quasi-mapping mode^61^. The index was built from the Ensembl GRCh38 transcriptome with a k-mer length of 31. Bias correction for sequence-specific and GC content was enabled, and mapping validation was performed with selective alignment enabled. Samples with fewer than 5% uniquely mapped reads were excluded from all downstream analyses to ensure robust quantification.

The resulting raw (unnormalized) gene count matrix was analyzed using DESeq2 v1.44.0 in R^62^. DESeq2 performed normalization using its median-of-ratios method, estimated dispersion parameters, and fitted a negative binomial generalized linear model for each gene. *P*-values were adjusted for multiple testing using the Benjamini–Hochberg false discovery rate (FDR) method. Functional enrichment was performed with the enricher module from gseapy v1.1.5^63,64^. KEGG gene sets were used as the reference pathway database. The enrichment method calculated a hypergeometric test for overlap significance and adjusted *p*-values via the Benjamini–Hochberg procedure, with enriched pathways defined as those having adjusted *p* < 0.05.

The Fast Task-driven Integrative Network Inference for Tissues (ftINIT) algorithm^65^ was employed to reconstruct context-specific genome-scale metabolic models from the transcriptomics dataset. Transcript expression levels were quantified as Transcripts Per Million (TPM). For each experimental group, the average TPM value across all biological replicates was calculated and used as the input expression profile for the reconstruction process. To filter out low-abundance transcripts, a minimum expression threshold of TPM ≥ 1 was applied; genes with average TPM values below this threshold were excluded from integration into the model. The reconstruction process incorporated tissue-specific expression data into the generic human metabolic network framework to produce context-specific models suitable for simulation. FBA was performed using ATP hydrolysis as the objective function, formulated to maximize ATP production potential under steady-state constraints. The optimization problem was solved using the solveLP function implemented in the RAVEN toolbox v2.10.1^66^ in MATLAB. The linear programming solver employed was the default solver configured within RAVEN (GLPK), operating with a feasibility tolerance of 1e-6 and optimality tolerance of 1e-6, ensuring high numerical precision.

### Statistics and reproducibility

Statistical analysis other than RNA-seq-based analysis was performed using GraphPad Prism 8 (GraphPad Software Inc.) Three individual infection experiments were performed using three different hiPSC lines, performed independently per hiPSC line in duplicates. Data are represented as the mean ± SD. In the corresponding figure legend, the specific statistical test performed for each analysis is indicated.

Immunocytochemistry figure preparation was done using LAS-X 3D software (Leica Microsystems) and ImageJ 1.50I. Flow cytometry data were visualized in histograms using FCS Express and Adobe Illustrator 2023. Bar graphs were made using Graphpad Prism 10.

### Reporting summary

Further information on research design is available in the Nature Portfolio Reporting Summary linked to this article.

## Supplementary information

Supplementary Information

Reporting Summary

## Data Availability

The raw data supporting the conclusions of this article are available in Figshare 10.6084/m9.figshare.31275061. The RNA-seq data have been deposited in the NCBI Sequence Read Archive (SRA) with accession no PRJNA1420755.

## Code availability

The code is available at https://github.com/SystemsVirology/HIV_Reservoir_Omics.
