# Supplementary material for: Microglia cause HIV-induced transcriptional and metabolic changes in human neural organoids
Source: Commun Biol. 2026 Mar 19;9:436. doi: 10.1038/s42003-026-09864-9 (PMC13021996; doi:10.1038/s42003-026-09864-9)
Supplement: Supplementary file 1 — Supplementary Information [file 42003_2026_9864_MOESM1_ESM.pdf]

Supplementary information

Supplementary Figure 1

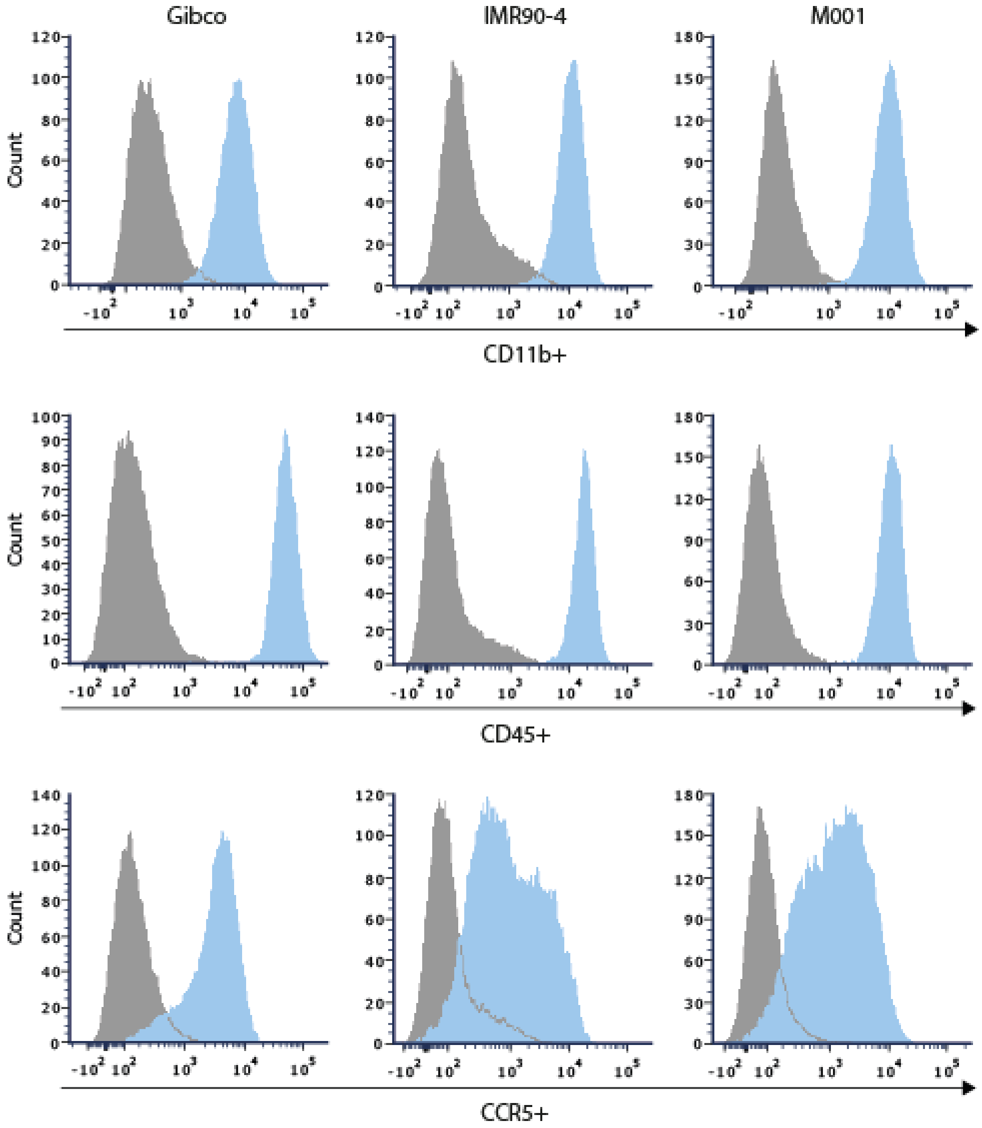

Supplementary Figure 1. Microglial marker expression of microglia generated from three human induced pluripotent cell lines (Gibco, IMR90-4, M001) measured by FACS (blue) compared to the unstained control (grey). Quality control was performed by flow cytometry as recommended by the manufacturer, using CD45 PerCP Cy5.5, and Cd11b AF488. CCR5 marker expression was confirmed using primary antibody CCR5 PE Clone 2D7. All samples showed >80% positivity in number of cells for each of the markers analysed.

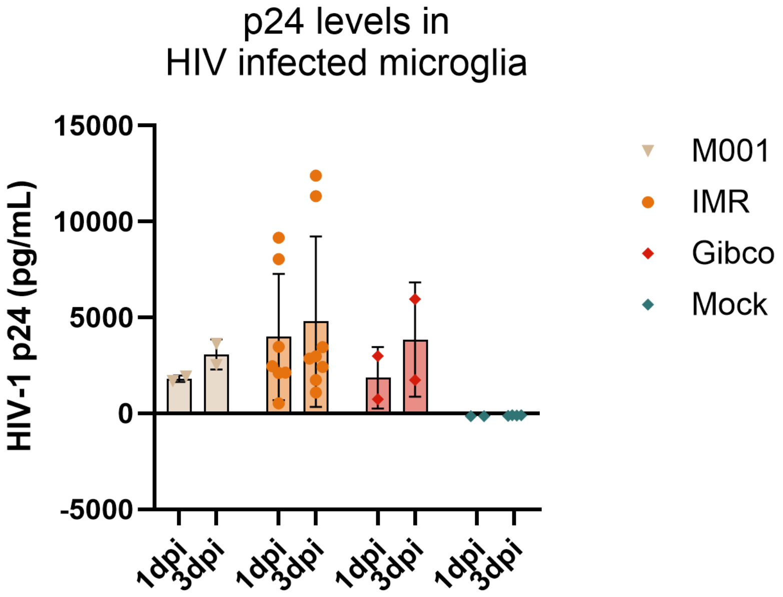

Supplementary Figure 2. HIV-1 p24 levels detected intracellular in microglia1 and 3 days post inoculation (1dpi and 3dpi).ELISA data showing detectable levels (pg/mL) of HIV-1 p24 in microglia generated from three distinct iPSC donors (M001, IMR90, and Gibco).

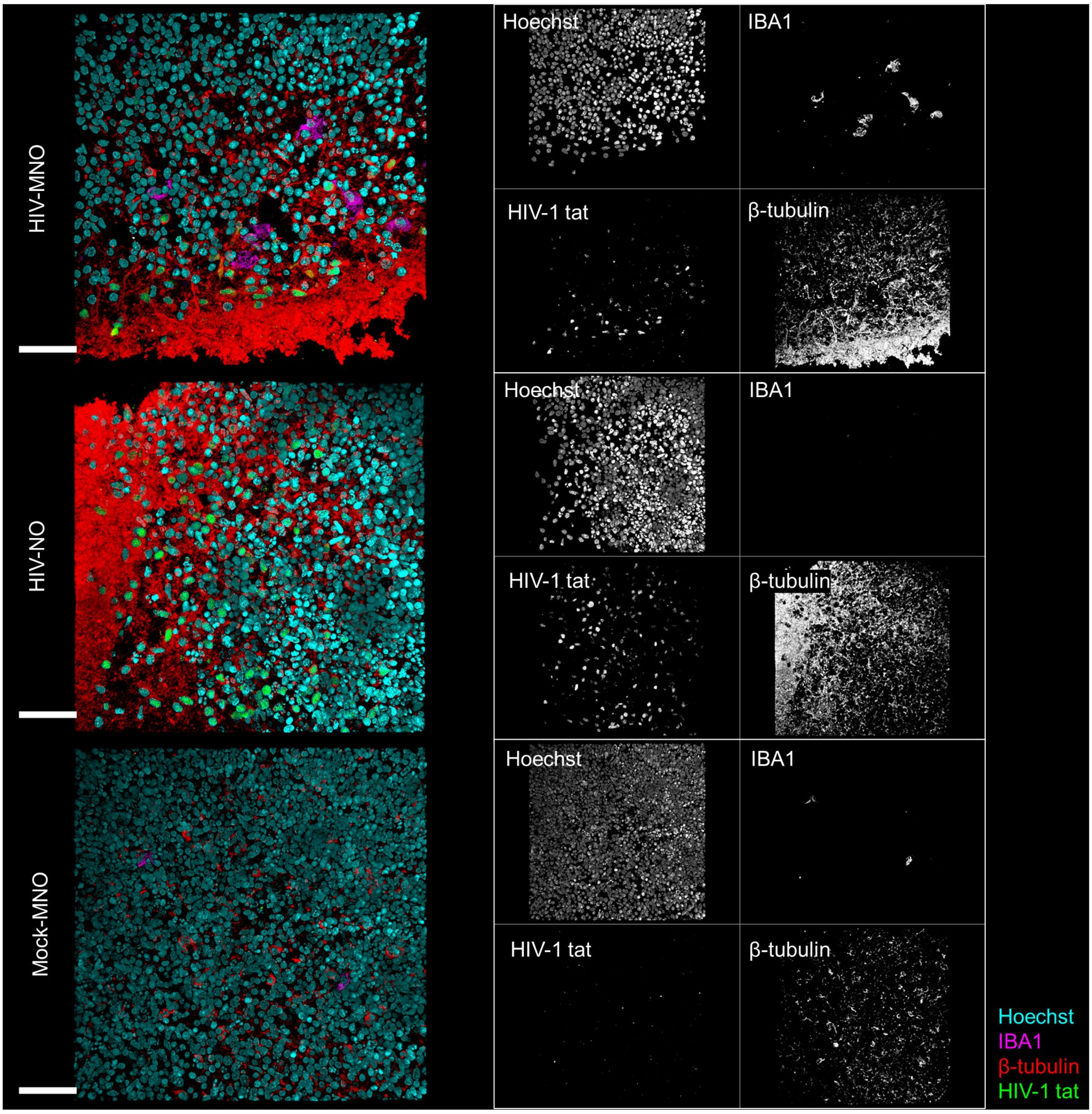

Supplementary Figure 3. Tat observed in MNO-infected and NO-infected.

Immunofluorescent staining shows both neural organoids that were infected with and without the presence of microglia show presence of tat. Nuclei (Hoechst), Microglia (Iba1) Neurons (β-tubulin), and HIV-1 tat protein (tat). Scale bar = 300μm.

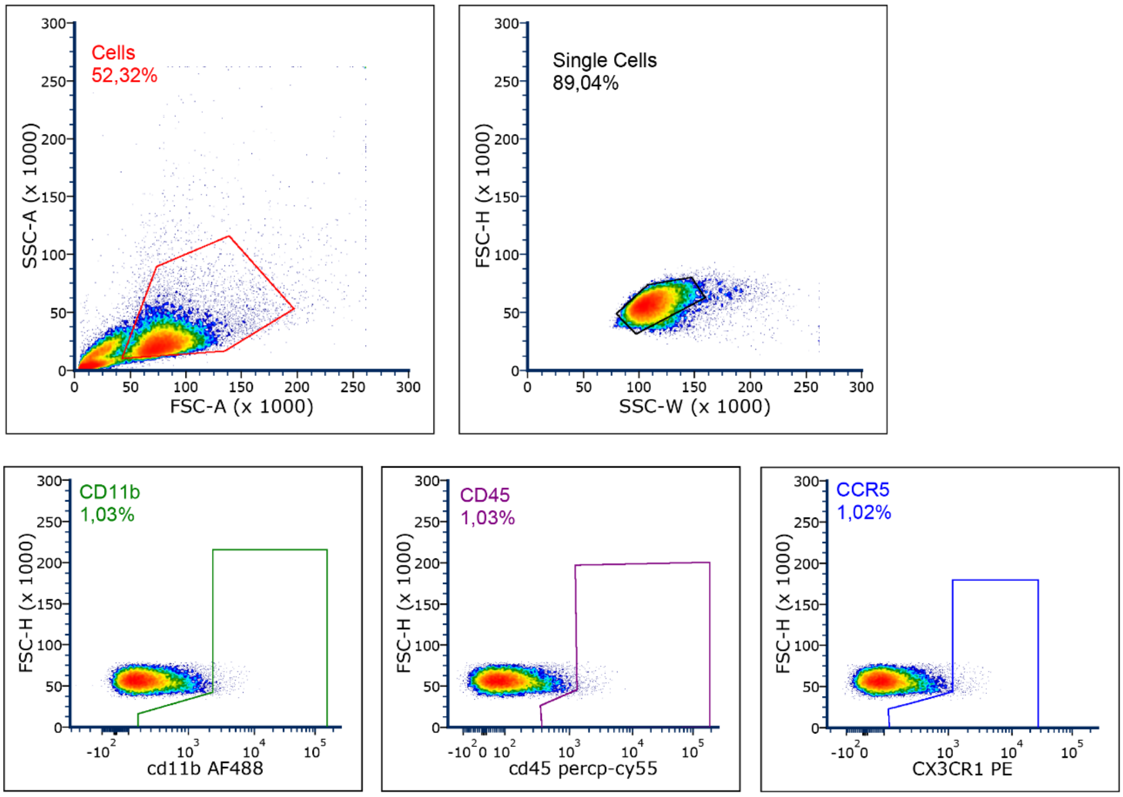

Supplementary Figure 4 – gating strategy microglia flow cytometry analysis. Debris in the sample was excluded by gating in the FSC-A SSC-A plot. Then, Single cell population was determined using gating in the SSC-W and FSC-H plot. From here, positive populations were determined using a mock channel and the respective fluorescent channel of interest. Background signal was determined using unstained controls.

Supplementary Table 1 – Antibody list

Antigen (abbreviation)	Origin	Dilution factor	Company	Catalog number
HIV1 p24 [P131]	Rabbit	1:400	Abcam	ab32352
HIV-1 core antigen-RD1, KC57	Mouse	1:400	Beckman Coulter	6604667
HIV-1 Tat-SF1 (C-4)	Mouse	1:100	Santa Cruz Biotechnology	sc-514351
Special AT-rich sequence-binding protein 2 (SATB2)	Mouse	1:400	Abcam	ab92446
IBA1	rabbit	1:500	Invitrogen	PA5-27436
β-III tubulin	rabbit	1:500	Invitrogen	PA5-86069
Glial fibrillary acidic protein (GFAP)	Goat	1:500	Abcam	Ab53554

Secondary Antibody (origin)	Secondary Antibody (origin)	Dilution factor	Company	Catalog number
Anti mouse Alexa Fluor 546 (donkey)	Donkey	1:750	Thermo Fisher	A10036
Anti rabbit Alexa Fluor 680 (donkey)	Donkey	1:750	Abcam	Ab175772
Anti goat Alexa Fluor 594 (donkey)	Donkey	1:750	Thermo Fisher	A11058

Supplementary Table 2 – RT-qPCR primer sets

Target	Forward sequence (5’-3’)	Reverse sequence (5’-3’)
HIV-1 Gag	CAGCAATCAGGTCAGCCAAAATTAC	CTTCTACTACTTTTACCCATGC
HIV-1 Pol	CTTCTAAATGTGTACAATCTAGTTGCC	TGATTTTAACCTGCCACCTGTAGTAG
RPLP0	TGGTCATCCAGCAGGTGTTCGA	ACAGACACTGGCAACATTGCGG
RPLP2	TCTTGGACAGCGTGGGTATCGA	CAGCAGGTACACTGGCAAGCTT
