# Supplementary material for: Microglia cause HIV-induced transcriptional and metabolic changes in human neural organoids
Source: Commun Biol. 2026 Mar 19;9:436. doi: 10.1038/s42003-026-09864-9 (PMC13021996; doi:10.1038/s42003-026-09864-9)
Supplement: Supplementary file 2 — Reporting Summary [file 42003_2026_9864_MOESM2_ESM.pdf]

nature portfolio

Corresponding author(s): Ujjwal Neogi

Last updated by author(s): Mar 3, 2026

Reporting Summary

Nature Portfolio wishes to improve the reproducibility of the work that we publish. This form provides structure for consistency and transparency in reporting. For further information on Nature Portfolio policies, see our Editorial Policies and the Editorial Policy Checklist.

Statistics

For all statistical analyses, confirm that the following items are present in the figure legend, table legend, main text, or Methods section.

n/a	Confirmed	
☐	☒	The exact sample size (*n*) for each experimental group/condition, given as a discrete number and unit of measurement
☐	☒	A statement on whether measurements were taken from distinct samples or whether the same sample was measured repeatedly
☐	☒	The statistical test(s) used AND whether they are one- or two-sided<br>*Only common tests should be described solely by name; describe more complex techniques in the Methods section.*
☒	☐	A description of all covariates tested
☒	☐	A description of any assumptions or corrections, such as tests of normality and adjustment for multiple comparisons
☒	☐	A full description of the statistical parameters including central tendency (e.g. means) or other basic estimates (e.g. regression coefficient) AND variation (e.g. standard deviation) or associated estimates of uncertainty (e.g. confidence intervals)
☐	☒	For null hypothesis testing, the test statistic (e.g. *F*, *t*, *r*) with confidence intervals, effect sizes, degrees of freedom and *P* value noted<br>*Give P values as exact values whenever suitable.*
☒	☐	For Bayesian analysis, information on the choice of priors and Markov chain Monte Carlo settings
☒	☐	For hierarchical and complex designs, identification of the appropriate level for tests and full reporting of outcomes
☒	☐	Estimates of effect sizes (e.g. Cohen's *d*, Pearson's *r*), indicating how they were calculated

Our web collection on statistics for biologists contains articles on many of the points above.

Software and code

Policy information about availability of computer code

Data collection	CFX Connect Real-Time PCR Detection System (Bio-Rad) using software CFX Maestro 1.1, Leica LAS AF Software (Leica Microsystems), LAS-X 3D software (Leica Microsystems) and ImageJ 1.50I, GraphPad Prism 8 (GraphPad Software Inc.)
Data analysis	*Provide a description of all commercial, open source and custom code used to analyse the data in this study, specifying the version used OR state that no software was used.*

For manuscripts utilizing custom algorithms or software that are central to the research but not yet described in published literature, software must be made available to editors and reviewers. We strongly encourage code deposition in a community repository (e.g. GitHub). See the Nature Portfolio guidelines for submitting code & software for further information.

Data

Policy information about availability of data

All manuscripts must include a data availability statement. This statement should provide the following information, where applicable:

- Accession codes, unique identifiers, or web links for publicly available datasets
- A description of any restrictions on data availability
- For clinical datasets or third party data, please ensure that the statement adheres to our policy

The raw data supporting the conclusions of this article are available in Figshare DOI: 10.6084/m9.figshare.31275061. The RNA-seq data have been deposited in NCBI Sequence Read Archive (SRA) with accession no PRJNA1420755.

Research involving human participants, their data, or biological material

Policy information about studies with human participants or human data. See also policy information about sex, gender (identity/presentation), and sexual orientation and race, ethnicity and racism.

Reporting on sex and gender	n.a.
Reporting on race, ethnicity, or other socially relevant groupings	n.a.
Population characteristics	n.a.
Recruitment	n.a.
Ethics oversight	n.a.

Note that full information on the approval of the study protocol must also be provided in the manuscript.

Field-specific reporting

Please select the one below that is the best fit for your research. If you are not sure, read the appropriate sections before making your selection.

☒ Life sciences ☐ Behavioural & social sciences ☐ Ecological, evolutionary & environmental sciences

For a reference copy of the document with all sections, see nature.com/documents/nr-reporting-summary-flat.pdf

Life sciences study design

All studies must disclose on these points even when the disclosure is negative.

Sample size	Three individual infection experiments were performed using three different hiPSC lines, performed independently per hiPSC line in duplicates.
Data exclusions	no data exclusions
Replication	infection of microglia was analysed repeatedly within and between stem cell donors to verify infectivity results are consistent in between and within stem cell donors. This is now added in Supplementary Figure 2.
Randomization	n.a.
Blinding	n.a.

Reporting for specific materials, systems and methods

We require information from authors about some types of materials, experimental systems and methods used in many studies. Here, indicate whether each material, system or method listed is relevant to your study. If you are not sure if a list item applies to your research, read the appropriate section before selecting a response.

Materials & experimental systems

n/a	Involved in the study	
☐	☒	Antibodies
☐	☒	Eukaryotic cell lines
☒	☐	Palaeontology and archaeology
☒	☐	Animals and other organisms
☒	☐	Clinical data
☒	☐	Dual use research of concern
☒	☐	Plants

Methods

n/a	Involved in the study	
☒	☐	ChIP-seq
☐	☒	Flow cytometry
☒	☐	MRI-based neuroimaging

Antibodies

Antibodies used	please find details in Supplementary_Table1 en 2

Validation	All antibodies used were commercially available and are broadly used. No custom antibodies were used for this manuscript.

Eukaryotic cell lines

Policy information about cell lines and Sex and Gender in Research

Cell line source(s)	PHA-stimulated human Peripheral Blood Mononuclear Cells (PBMCs) were isolated from buffy coats of healthy donors (Sanquin) by Lymphoprep (Axis-Shield) gradient
Authentication	positive selection using anti CD45+ antibodies by Magnetic sorting
Mycoplasma contamination	Mycoplasma tests are routinely performed in our laboratory. No positive results were obtained during the procedure of generating this manuscript.
Commonly misidentified lines (See ICLAC register)	n.a.

Plants

Seed stocks	n.a.
Novel plant genotypes	n.a.
Authentication	n.a.

Flow Cytometry

Plots

Confirm that:

☒ The axis labels state the marker and fluorochrome used (e.g. CD4-FITC).

☒ The axis scales are clearly visible. Include numbers along axes only for bottom left plot of group (a 'group' is an analysis of identical markers).

☒ All plots are contour plots with outliers or pseudocolor plots.

☒ A numerical value for number of cells or percentage (with statistics) is provided.

Methodology

Sample preparation	Microglia cells were harvested and washed with PBS followed by centrifugation at 300g for 5 min. and fixed and permeabilized using 250 µL of Cytofix/Cytoperm solution (BD, 554714) for 20 min. at 2 - 8°C. Cells were washed twice with 1mL BSA Stain Buffer (BD, 554657), centrifuged at 300g for 5 min, and blocked using 2µl FC-block (BD, 564220) per sample for 5 min. at 2 - 8°C. Cells were stained with a conjugated primary antibody CD11b-488 (60040AD.1, STEMCELL Technologies™), CD45 (60018PS.1 (STEMCELL Technologies™) and CCR5 (CCR5 PE Clone 2D7, BD Pharmingen, 555993) at a dilution of 1:25 in a total volume of 25ul and incubated for 30 min. at 2 - 8°C followed by two washes with 1mL BSA Stain Buffer (BD, 554657). Samples were resuspended in 100ul BSA Stain Buffer and kept in the dark at 2 - 8°C until analysis on the flow cytometer (BD FacsCanto ll).
Instrument	BD FacsCanto ll
Software	FCS Express v6
Cell population abundance	The sample contained cells and debris, which was excluded by gating in the FSC-A SSC-A plot. Then, Single cell population was determined using gating in the SSC-W and FSC-H plot. From here, positive populations were determined using a mock channel and the respective fluorescent channel of interest. Background signal was determined using unstained controls.
Gating strategy	The sample contained cells and debris, which was excluded by gating in the FSC-A SSC-A plot. Then, Single cell population was determined using gating in the SSC-W and FSC-H plot. From here, positive populations were determined using a mock channel and the respective fluorescent channel of interest. Background signal was determined using unstained controls. This is now included as Sup. Figure 4

☒ Tick this box to confirm that a figure exemplifying the gating strategy is provided in the Supplementary Information.
